# Increased Prevalence of Intermittent Rhythmic Delta or Theta Activity (IRDA/IRTA) in the Electroencephalograms (EEGs) of Patients with Borderline Personality Disorder

**DOI:** 10.3389/fnbeh.2016.00012

**Published:** 2016-02-23

**Authors:** Ludger Tebartz van Elst, Max Fleck, Susanne Bartels, Dirk-Matthias Altenmüller, Andreas Riedel, Emanuel Bubl, Swantje Matthies, Bernd Feige, Evgeniy Perlov, Dominique Endres

**Affiliations:** ^1^Section for Experimental Neuropsychiatry, Department for Psychiatry and Psychotherapy, University Medical Center FreiburgFreiburg, Germany; ^2^Department of Neurosurgery, Freiburg Epilepsy Center, University Medical Center FreiburgFreiburg, Germany; ^3^Department for Psychiatry and Psychotherapy, Saarland University Medical CenterHomburg, Germany

**Keywords:** IRDA, IRTA, local area network inhibition, EEG, borderline personality disorder

## Abstract

**Introduction:** An increased prevalence of pathological electroencephalography (EEG) signals has been reported in patients with borderline personality disorder (BPD). In an elaborative case description of such a patient with intermittent rhythmic delta and theta activity (IRDA/IRTA), the BPD symptoms where linked to the frequency of the IRDAs/IRTAs and vanished with the IRDAs/IRTAs following anticonvulsive therapy. This observation raised a question regarding the prevalence of such EEG abnormalities in BPD patients. The aim of this retrospective study was to identify the frequency of EEG abnormalities in a carefully analyzed psychiatric collective. Following earlier reports, we hypothesized an increased prevalence of EEG abnormalities in BPD patients.

**Participants and Methods:** We recruited 96 consecutive patients with BPD from the archive of a university clinic for psychiatry and psychotherapy, and compared the prevalence of EEG abnormalities to those of 76 healthy controls subjects. The EEGs were rated by three different blinded clinicians, including a consultant specializing in epilepsy from the local epilepsy center.

**Results:** We found a significant increase in the prevalence of IRDAs and IRTAs in BPD patients (14.6%) compared to the control subjects (3.9%; p = 0.020).

**Discussion:** In this blinded retrospective case-control study, we were able to confirm an increased prevalence of pathological EEG findings (IRDAs/IRTAs only) in BPD patients. The major limitation of this study is that the control group was not matched on age and gender. Therefore, the results should be regarded as preliminary findings of an open uncontrolled, retrospective study. Future research performing prospective, controlled studies is needed to verify our findings and answer the question of whether such EEG findings might predict a positive response to anticonvulsive pharmacological treatment.

## Introduction

Electroencephalography (EEG) represents a method by which the functional integrity of the brain can be investigated with high temporal, but poor spatial, resolution. The discovery of this method by Hans Berger (Berger, 1969; Shipton, 1975) marks the advent of modern biological psychiatry. This paper focuses on EEG abnormalities in patients with Borderline Personality Disorder (BPD).

The concept of BPD was developed in the 1970s and was introduced as a specific diagnostic category in DSM-III in 1980 (Goldstein, 1983). It is characterized by the core features of emotional instability, impulsivity, and instability of self-image and interpersonal relationships (Lieb et al., 2004). Since, episodic dissociative phenomena that are often related to very aversive states of inner tension also belong to the features of BPD and because this is reminiscent of complex partial epileptic seizures, a pathophysiological link to epilepsy has been discussed in the past (Harris et al., 2002; Williams et al., 2006; Tebartz van Elst et al., 2011). Further, there has been discussion as to whether patients with specific epilepsy syndromes such as juvenile myoclonic epilepsy might develop BPD features more often than people with other forms of epilepsy or healthy controls (de Araujo Filho and Yacubian, 2013).

Early EEG studies found an increased prevalence of generally diffuse EEG pathology in BPD patients (De La Fuente et al., 1998; Reeves et al., 2003). In a meta-analysis by Shelley et al. (2008), the respective prevalence rates were estimated to be between 5.8 and 46%. However, most of the summarized studies were hampered by methodological problems, the samples sizes were rather small or the EEG ratings had not been done in a blinded way.

In this context, we recently published the case of a patient with BPD and the EEG pathology of intermittent rhythmic delta and theta activity (IRDA and IRTA) with very severe dissociative states of inner tension and autoaggressive behavior. Anticonvulsive treatment with valproate led to remission of clinical symptoms and a significant reduction in the frequency of IRTAs. Based on these observations, we have put forward the hypothesis of “local area network inhibition” (LANI hypothesis), which holds that non-ictal, paroxysmal neuronal activity (like sharp waves, polyspikes, spike-wave complexes [SWCs], IRDAs, IRTAs) induces inhibitory adaptation processes in local networks in an attempt by the brain to maintain the excitatory-inhibitory homeostasis of the affected networks. Rare and distributed trigger activity may induce sub-threshold inhibition, which can fade away without producing neuropsychiatric symptoms, while frequent and focal trigger activity could eventually induce supra-threshold LANI, which then results in neuropsychiatric symptoms depending on the affected cerebral site or circuit (Tebartz van Elst et al., 2011).

IRDAs were originally described by Cobb in 1945 (Cobb, 1945). According to the local distribution, IRDAs can be categorized as frontal IRDAs (FIRDAs), temporal IRDAs (TIRDAs), and occipital IRDAs (OIRDAs). Without any local specification, it is appropriate to simply refer to IRDAs. They are regarded as a pathological EEG pattern of unknown pathophysiological significance (Brigo, 2011). Within the framework of the LANI model, we hypothesized that IRDAs may have the pathophysiological potential to induce adaptive homeostatic processes, potentially leading to functional alternations of the affected neuronal networks. This assumption is, for example, supported by observations that such EEG phenomena are linked to complex neuropsychiatric symptoms in the context of limbic encephalitis (van Vliet et al., 2012) or to symptoms of migraine (Kakisaka et al., 2014).

The LANI model could explain the pathophysiology of some symptoms in a subgroup of patients with BPD (i.e., those who present with EEG abnormalities like IRDAs or IRTAs). One might put forward the hypothesis, that—as in the case mentioned above (Tebartz van Elst et al., 2011)—such patients could well respond to therapy with anticonvulsants. Following this line of thought, it is remarkable that the therapeutic efficacy of anticonvulsants in cases of BPD has been described for a number of different substances (Bellino et al., 2005; Loew et al., 2006; Stoffers et al., 2010; Vita et al., 2011; Ripoll, 2012). Given these considerations, it is noteworthy that in clinical practice many patients with BPD do not receive a diagnostic work-up that includes a thorough EEG investigation. However, before calling for such laborious procedures, it is important to clarify how often such EEG phenomena can be expected in unselected patients with BPD.

### Rationale for our study

In our clinic, we have a ward that specializes in providing in-patient treatment for BPD patients through employing dialectic behavioral therapy (Lynch et al., 2007). Even though the focus of this ward is psychotherapy, we traditionally perform EEG analyses for all hospitalized patients of our clinic. The current clinical study takes advantage of this fact in that it enables us to retrospectively analyze the frequency of relevant EEG pathologies in these patients. Thus, the aim of this retrospective study was to clarify the frequency of EEG abnormalities (i.e., SWCs, IRDAs, and IRTAs) in BPD patients. Based on the findings of earlier studies (Shelley et al., 2008), we hypothesized an increased prevalence of EEG abnormalities in BPD patients.

## Participants and methods

The study received approval from the Ethics Committee of the University of Freiburg (EK-Fr 233/14).

### Composition of the patient group

We included patients suffering from BPD who had been admitted to our hospital for in-patient treatment between 2001 and 2011. Since visual, high-quality EEG analysis is very time consuming, we were not able to include all datasets. In the absence of reliable and expectable prevalence rates from earlier controlled studies, we decided to adopt a pragmatic approach and included the first 100 consecutive patients who fulfilled all inclusion and exclusion criteria. Due to *post-hoc* information, we had to exclude four BPD patients, which led to 96 BPD patients being included.

The BPD patients were diagnosed according to the standards of our specialized unit, i.e., the Borderline diagnosis was established by senior consultant psychiatrists based on a detailed structured psychiatric interview (Structured Clinical Interview for DSM-IV, SCID I and II; First et al., 1996, 1997) that integrated common psychiatric and somatic differential diagnoses as well as the patients' medical histories. We included more female patients (93 out of 96) because our in-patient treatment is especially designed for female BPD patients.

Patients with known comorbid organic psychiatric disorders, psychotic disorders, or other personality disorders were excluded from the study. We also excluded patients with any neurological disorder, a history of birth complications, febrile convulsions or encephalitic disease in the past. A family history of epilepsy also led to exclusion from our study. Antiepileptic medication can reduce epileptic EEG patterns (Duncan, 1987) and so might lead to the underestimation of EEG abnormalities, while clozapine is known to be the most proconvulsive medication and can lead to an overestimation of EEG abnormalities (Welch et al., 1994; Meyer, 2004; Alper et al., 2007). Therefore, we also excluded patients taking anticonvulsant medication or the proconvulsant drug clozapine. For patients who received more than one EEG, we selected the initial one for this study.

### Composition of the control group

In our daily clinical practice, we do not analyze the EEGs of healthy controls. Therefore, we included controls from an earlier, large EEG study conducted in-house. Again, any psychiatric or neurological diagnoses lead to exclusion. Nicotine consumption was not an exclusion criterion. We were able to include all 76 datasets for which electronic records were available (Feige et al., 2008). In addition, we compared the findings of our BPD sample to figures from the literature. Based on published data, EEG abnormalities in healthy controls who received neurological as well as psychiatric assessments were found in about 0.5% of cases (study of 13,658 trainee pilots; Gregory et al., 1993). For the statistical analysis, we conservatively assumed EEG abnormalities in 1% of the general population (Shelley et al., 2008).

### EEG reading and classification

**Figure 1:**
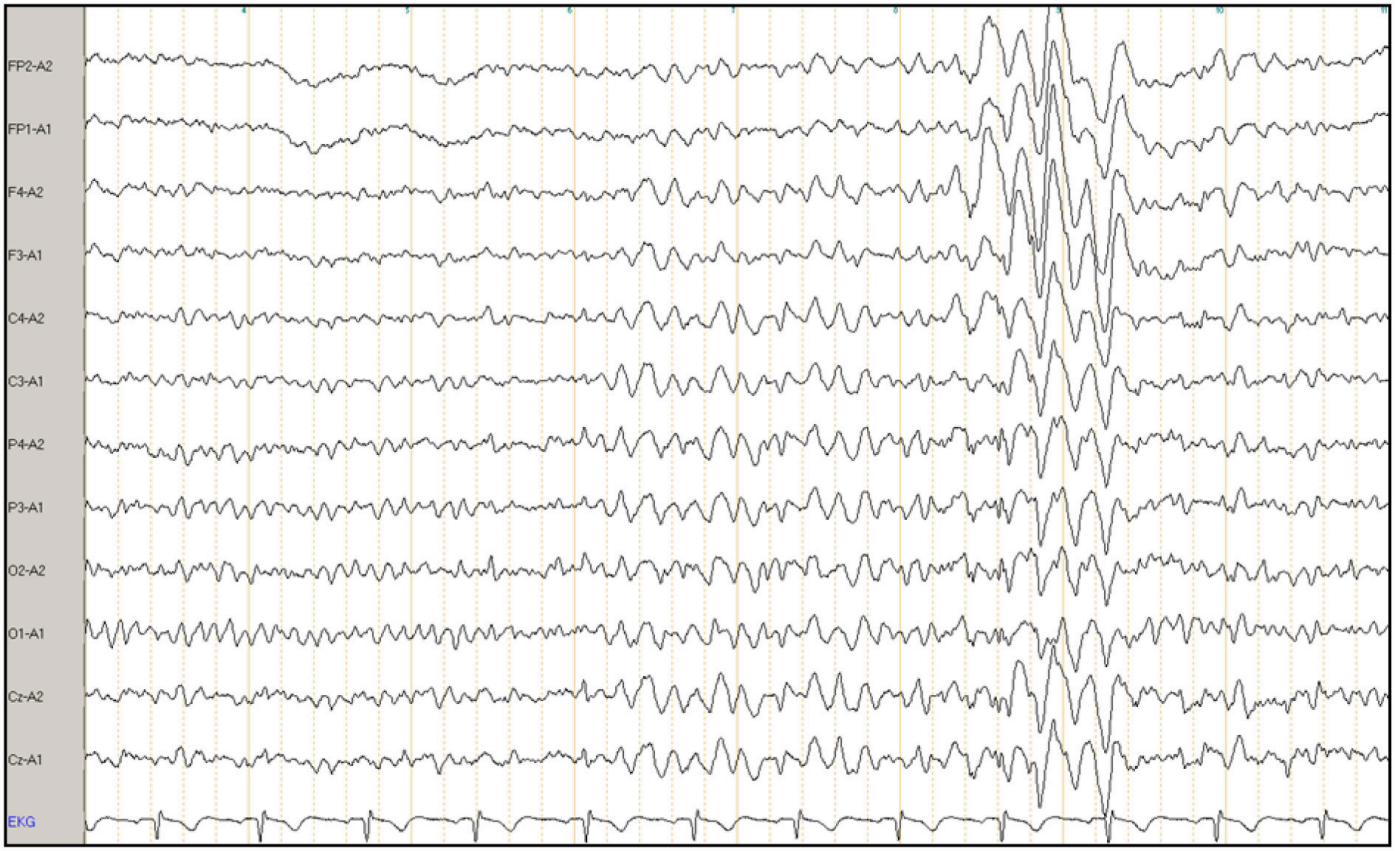
**Example of a clinical electroencephalogram (EEG) of a patient with borderline personality disorder**. The X-axis illustrates intermittent rhythmic theta activity (IRTA), and Y-axis shows a referential montage to the ears. FP, frontoparietal; A, ear; F, frontal; C, central; P, parietal; O, occipital; Cz, central zero; EKG, electrocardiography.

All EEGs were recorded using the international 10/20 system for 20 min, including a hyperventilation phase of more than 3 min, which was used as a provocation method. Sintered Ag-AgCl bridge electrode impedances were kept below 5 kOhm. Signals were acquired using a Schwarzer 25-channel USB amplifier, filtered between 0.07 and 100 Hz, sampled with a rate of 256 Hz and continuously stored on disc for later analysis. Neurofile® software was used for visual EEG analysis following typical clinical standards. Longitudinal rows were used as standard montage. In cases of pathological findings in the bipolar longitudinal rows, we correlated abnormalities with bipolar transverse rows and reference electrodes. All EEGs were analyzed by the same blinded trained expert rater (MF), who identified all normal EEGs in a first step. The abnormal and all nebulous EEG findings were reevaluated in a second step by a board certified neurologist (SB). All EEGs that were deemed abnormal following this second step were rated by a board certified consultant epileptologist from the local university epilepsy center (DMA). All raters were blinded throughout the diagnostic process. Final diagnosis was established as a consensus diagnosis of all raters. All abnormalities were documented (i.e., each IRDA, IRTA, and SWC). Figure 1 shows an exemplary EEG with IRTA. To define clear outcome criteria and to avoid the overestimation of pathological EEGs based on diffuse slowing due to drowsiness or hyperventilation, we took care to exclude such phenomena of physiological slowing and did not rate any unclear EEG slowing as a pathological phenomenon.

### Demographic and psychometric data

All demographic and psychometric information was assessed using our clinic's electronic documentation system. This data comprised demographic data, psychopathological symptoms following the standardized AMDP system (Arbeitsgemeinschaft für Methodik und Dokumentation in der Psychiatrie, www.amdp.de), medical and psychiatric history, medication, and social information. Only patients with complete documentation were included.

### Data handling and statistical analysis

All EEG, demographic, and psychometric information was carefully evaluated and entered into a data bank using the Statistical Package for the Social Sciences (SPSS 20). Age was compared using two-sided independent-sample *t*-tests, while gender was analyzed using Pearson's two-sided *X*^2^-test. The prevalence of EEG abnormalities between groups was compared using Pearson's two-sided *X*^2^-test. We compared the prevalence rates of the BPD patients with both our own and a historical control group. Moreover, we compared age matched groups, after the exclusion of the oldest control subjects. In addition, we compared only female patients and female controls. To exclude any effects of medication, we compared the unmedicated BPD patients with the control group. A *p* < 0.05 served as the criterion of significance.

## Results

### Demographic data

Table 1 summarizes the clinical and demographic data of our patients and the control group. The BPD patients turned out to be significantly younger than the members of the control group. Also, since the control group was not specifically generated to be matched with this study, it consisted of a significantly higher number of male subjects.

**Table 1:** **Demographic and clinical characteristics of borderline personality disorder and control group**.

	**Borderline personality disorder (*n* = 96)**	**Control group (*n* = 76)**	**Statistics**
Age	27.0 ± 6.9	37.8 ± 10.8	*t* = −7.7, *df* = 121.1, *p* ≤ 0.001
Gender	93 F:3 M	38 F:38 M	*X*^2^ = 51.3, *df* = 1, *p* ≤ 0.001
**SCHOOL EDUCATION**			
No degree	4 (4.2%)	Not available	
Low degree	27 (28.1%)	Not available	
Medium degree	35 (36.5%)	Not available	
High degree	28 (29.2%)	Not available	
Unknown	2 (2.1%)	Not available	
**PROFESSIONAL DEGREE**			
None/semiskilled	53 (55.2%)	Not available	
Vocational training	31 (32.3%)	Not available	
University degree	10 (10.4%)	Not available	
Unknown	2 (2.1%)	Not available	
**MEDICATION**			
Medication on discharge	55 (57.3%)	Unmedicated	
No medication	41 (42.7%)	Unmedicated	
ABNORMAL PSYCHOPATHOLOGICAL FINDINGS^*^			
Attention and memory	50 (52.1%)	Not inquired	
Formal thought disorder	18 (18.8%)	Not inquired	
Fear and compulsion	26 (27.1%)	Not inquired	
Affectivity	86 (89.6%)	Not inquired	
Energy and psychomotor domain	51 (53.1%)	Not inquired	
Circadian rhythm	32 (33.3%)	Not inquired	
Suicidal tendency	17 (17.7%)	Not inquired	
**PSYCHIATRIC COMORBIDITY**			
Psychiatric overall comorbidity	60 (62.5%)	Excluded	
ADHD	29 (30.2%)	Excluded	
Major Depression	Current: 25 (26%) Remitted: 5 (5.2%)	Excluded	
Eating disorder	11 (11.5%)	Excluded	
Adaptation disorder	4 (4.2%)	Excluded	
Others^**^	12 (12.5%)	Excluded	

* Documented on discharge;

** *Phobia (in 3 patients), alcohol abuse (3), obsessive compulsive disorder (2), dysthymia (1), bipolar disorder (1), tic disorder (1), somatoform disorder (1). F, female; M, male; ADHD, attention deficit hyperactivity disorder*.

### Prevalence of EEG abnormalities

Only IRDAs and IRTAs were found in the patient and control groups. No patient showed clear-cut epileptiform potentials such as sharp waves, polyspikes, or spike-wave complexes. Still, the BPD patients demonstrated significantly higher rates of EEG abnormalities (*X*^2^ = 5.4, *df* = 1, *p* = 0.020). When comparing the rate of EEG abnormalities to the 1% estimated from figures in the literature, this difference became even more significant (*X*^2^ = 12.8, *df* = 1, *p* ≤ 0.001).

When comparing only unmedicated BPD patients (*n* = 41) to all controls, we also found increased percentages of EEG abnormalities (*X*^2^ = 4.3, *df* = 1, *p* = 0.038; Table 2). Comparing only female BPD patients (*n* = 93, age: 27.1 ± 6.9) and female controls (*n* = 38; age: 40.1 ± 11.7; *p*_age_ ≤ 0.001), we find higher rates of EEG pathologies in the female patients (15.1 vs. 5.3% in the controls), showing a non-significant trend in the *X*^2^-test (*X*^2^ = 2.4, *df* = 1, *p* = 0.120). Due to the small sample size of male patients (*n* = 3), we have not compared the male groups. After excluding the oldest controls, we analyzed the age matched groups (96 BPD patients vs. 41 controls). Again, the EEG pathologies were higher in the BPD group (14.6 vs. 7.3% in the controls), which was not significant (*X*^2^ = 1.4, *df* = 1, *p* = 0.237).

**Table 2:** **EEG abnormalities in borderline personality disorder**.

	**Borderline personality disorder (*n* = 96)**	**Control group (*n* = 76)**	**Statistics (Pearson-*X*^2^-test)**
IRDAs/ IRTAs	14 (14.6%)	3 (3.9%)	*X*^2^ = 5.4; *df* = 1; ***p** = **0.020***
	**Unmedicated borderline personality disorder group (***n* = 41**)**	**Control group (***n* = 76**)**	
IRDAs/ IRTAs	6 (14.6%)	3 (3.9%)	*X*^2^ = 4.3; *df* = 1; ***p** = **0.038***
	**Borderline personality disorder (***n* = 96**)**	**Historical control group** (Gregory et al., 1993; Shelley et al., 2008)	
IRDAs/ IRTAs	14 (14.6%)	1%	*X*^2^ = 12.8; *df* = 1; ***p** ≤ **0.001***

*IRDAs, intermittent rhythmic delta activity; IRTAs, intermittent rhythmic theta activity*.

### Clinical characteristics of BPD patients with IRDAs/IRTAs

Table 3 summarizes the clinical characteristics of the 14 BPD patients with EEG abnormalities. All patients were female and suffered mostly from mental tension, self-injuries and dissociative symptoms. Five patients had attempted suicide. Six out of 14 patients were unmedicated, while eight patients were taking antidepressants. Depression was the most frequent comorbidity in patients with IRDAs/IRTAs. Three patients had a comorbid eating disorder.

**Table 3:** **Characterization of patients with IRDAs/IRTAs**.

**Nr**.	**Patient characteristics**	**Paroxysmal symptoms**	**Comorbidity**	**Medication**	**EEG-abnormalities**
1	21 years, female, trainee nurse	Dissociative symptoms, self injuries, suicidal tendency, several suicidal attempts	Depression, PTSD	None	IRDAs with frontal maximum
2	26 years, female, industrial clerk	Mental tension, difficulties controlling anger, self injuries, several suicidal attempts	Atypical bulimia nervosa	None	IRDAs with frontal maximum
3	28 years, female, commercial clerk	Affective instability, self injuries	Depression (currently remitted)	Citalopram	FIRTAs
4	22 years, female, media worker	Mental tension, self injuries, affective instability	None	None	IRTAs
5	19 years, female, no professional training	Mental tension, self injuries, dissociative symptoms, suicidal tendency, one suicidal attempt	Past alcohol abuse	None	IRTAs with fronto-central maximum
6	19 years, female, school for domestic science	Dissociative seizures, mental tension, self injuries	None	None	IRTAs
7	22 years, female, no professional training	Mental tension, self injuries	Mild depression, past substance abuse, two suicidal attempts	Venlafaxine	IRDAs with frontal maximum
8	25 years, female, no professional training	Dissociative states, mental tension, self injuries, three suicidal attempts	Anorexia nervosa, depression	Quetiapine, fluoxetine, benperidol	IRTAs with parieto-temporal maximum
9	22 years, female, trainee office clerk	Mental tension, self injuries, suicidal tendency, several suicidal attempts	Depression (currently remitted)	Venlafaxine, prothipendyl, chlorprothixene	FIRTAs
10	19 years, female, trainee hotel clerk	Mental tension, self injuries	None	None	FIRTAs
11	26 years, female, insurance clerk	Dissociative states, mental tension, suicidal tendency	Depression, ADHD, PTSD	Methylphenidate, fluspirilene	FIRTAs
12	25 years, female, insurance clerk	Mental tension, self injuries, dissociative states with depersonalization and derealization, suicidal tendency	Atypical bulimia nervosa, depression (currently remitted)	Citalopram, perazine	FIRTAs
13	34 years, female, profession unclear	Mental tension, self injuries, derealization, hallucinations	None	Risperidone, zopiclone, chlorprothixene, fluoxetine, tetrazepam	FIRTAs
14	27 years, female, office clerk	Dissociative symptoms, mental tension, self injuries, suicidal tendency	ADHD, abuse of alcohol	Venlafaxine, zopiclone	FIRTAs

*EEG, electroencephalogram; PTSD, Post-Traumatic Stress Disorder; ADHD, attention deficit hyperactivity disorder; IRDA, intermittent rhythmic delta activity; IRTA, intermittent rhythmic theta activity; FIRTA, frontal intermittent rhythmic theta activity*.

## Discussion

The main result of our study is that, among the BPD patients, there was a significant subgroup of 14.6% who displayed clear-cut EEG abnormalities in terms of IRDAs and IRTAs. In contrast to other studies, no epileptic patterns could be detected. However, due to the not exactly matched control group, our results should be regarded as only preliminary findings.

### Comparison with previous studies

Our finding is well within the range of EEG abnormalities in BPD patients reported in the review by Shelley and colleagues (5.8–46%; Shelley et al., 2008; Table 4). In our study, only IRDAs and IRTAs were found, as in some previous studies involving BPD patients (Snyder and Pitts, 1984; De La Fuente et al., 1998). Snyder and Pitts (1984) found significantly more abnormalities in 37 male BPD patients, mostly slow wave activity. De La Fuente et al. (1998) described diffuse slow activity in 40% of the mostly female patients in a collective of 20 patients. In a study by Archer et al. (1988), dysrhythmia was found in 31.3%, while in a study by Cornelius et al. (1986) it was detected in 18.8% of the BPD patients. Ogiso et al. (1993) depicted an association between positive spikes and high impulsivity. Our low overall prevalence rates might be explained by our strict inclusion and exclusion criteria, which excluded all patients with any evidence of organic comorbidities and relevant medication effects. Also, due to our desire to avoid an overestimation of pathological EEG and in contrast to many other studies in the literature, we did not rate unclear EEG slowing as pathological phenomena. Moreover, differences in the reference electrodes in earlier studies might have influenced the analysis process and findings.

**Table 4:** **Previous EEG findings in borderline personality disorder (following Boutros et al., 2003; Shelley et al., 2008)**.

**Study**	**N (PBD/ controls)**	**Gender patients (F/M)**	**Medication**	**Comorbidity**	**EEG-abnormalities**	**Characteristics**
Tebartz van Elst et al., 2011	1/0	1/0	None	None	Epileptiform discharges	Remission with valporate
De La Fuente et al., 1998	20/0	15/5	None	None current	Diffuse slow activity in 40%	More frequent in medicated patients
Ogiso et al., 1993	18/21	18/0	Anxiolytics, antipsychotics, antidepressants	Depression, substance abuse	Positive spikes in patients with high impulsivity; Wave and spike phantoms in patients with interpersonal relationship dysfunction	
Drake et al., 1992	6/0	Not reported	Not reported	Not reported	Normal findings in patients with pseudo-seizures	
Schmid et al., 1989	1/0	1/0	Antidepressant, antipsychotic	Depression	Normal	
Cowdry et al., 1985	39/20 (unipolar depressed patients)	36/3	Not reported	No current Axis I disorder, Axis II not reported	More frequent epileptiform discharges in BPD; mostly paroxysmal posterior sharp waves	
Messner, 1986	1/0	0/1	None	None	Focal temporal lobe slow-wave activity	
Archer et al., 1988	16/83 (10 with non-BPD personality disorders, 39 with dysthymic disorders, 34 with other mixed diagnosis)	Not reported	None	None	Dysrhytmics in 31.3%; 6.3% had spike and wave discharges	No significant differences compared with control groups; wake and sleep EEG
Cornelius et al., 1986	69/22 (non-BPD personality disorders)	52/17	None	None current	Dysrhythmias in 18.8% of BPD patients; severe abnormalities in 5.8%	No significant differences compared with non-BPD personality disorder group
Snyder and Pitts, 1984	37/31 (dysthymic disorder)	0/37	None	None	Significantly more abnormalities, mostly slow-wave activity	Only male patients

*BPD, borderline personality disorder; F, female; M, male; EEG, electroencephalogram*.

### Pathological EEG findings in healthy subjects

In order to judge whether or not such findings are relevant, it is important to clarify how often similar pathological EEG findings are obtained in the general population without BPD. Depending on the inclusion and exclusion criteria, EEG abnormalities were identified in 0.3–18.6%, showing a broad range of “normality” (Gregory et al., 1993; Boutros et al., 2005; Shelley et al., 2008). However, after the exclusion of possible contaminating factors, epileptiform dysrhythmia in healthy control groups can be assumed to account for less than 1% of cases (Shelley et al., 2008). Therefore, the authors of review articles regarding EEG abnormalities in control groups and in psychiatric collectives argue for stricter inclusion and exclusion criteria, such as those enforced in our study (Boutros et al., 2005; Shelley et al., 2008).

### Limitations

The major limitation of this study is that it is a retrospective analysis without adequate control group. Therefore, the results should basically be regarded as preliminary findings of an open uncontrolled study and statistical calculations should be regarded as only exploratory in nature. We cannot be sure about what is driving the difference in occurrence of EEG abnormalities in this sample. In any retrospective analysis of EEG patient collectives, it is difficult to generate suitable control groups because controls are not routinely investigated. Therefore, further and prospective studies are needed to confirm the findings raised in this paper.

For this study, we were able to use a control group of healthy participants who took part in an earlier project involving sleep research (Feige et al., 2008). However, this control group was smaller than our patient group and was not well matched with respect to gender and age. The BPD patients were younger than the controls and females were more common than in the control group. The control subjects did not receive a thorough neurological or psychiatric assessment. Earlier evidence from basic clinical EEG research illustrates that the less strictly the EEG control samples were defined in terms of neurological and psychiatric evaluations, the higher the resulting prevalence of EEG pathologies turned out to be (Tebartz van Elst and Perlov, 2013). In large cohorts of well investigated pilots who had received not only neurological but also internistic and psychiatric evaluations, pathological EEGs proved to be very rare, with a prevalence of between 0.3 and 0.6% (Thorner, 1942; Bennett, 1967; Gregory et al., 1993). In contrast, less well defined control samples produced prevalence figures for pathological EEG findings in 5–8% of cases (Iida et al., 1985; Okubo et al., 1993). In that sense, our control sample is comparable to the clinical samples published by Iida et al. (1985) and Okubo et al. (1993), with pathological findings in 3.9% of cases. Therefore, we would have detected false positive EEG pathologies in our control group.

Moreover, the effect of medication could not be completely eliminated since we were unable to include only unmedicated patients. We excluded patients who were currently taking the most proconvulsive (clozapine; Duncan, 1987; Meyer, 2004; Alper et al., 2007) and typical anticonvulsive medications (antiepileptics). However, other neuroleptics or antidepressants might have influenced our results. Neuroleptics (e.g., olanzapine) could reduce the seizure threshold to a lesser extent compared with clozapine; some antidepressants—mainly selective serotonin reuptake inhibitors—might have the opposite effect and actually increase the seizure threshold (Alper et al., 2007; Tebartz van Elst and Perlov, 2013).

However, six of the 14 BPD patients with pathological EEGs were unmedicated and nine were medicated. A total of 41 out of the 96 BPD patients were unmedicated. When restricting the analysis to these 41 unmedicated patients and comparing EEG abnormalities with our control group, the prevalence of pathological EEG findings was the same (14.6%). This means that our overall prevalence rates cannot be explained by a medication effect.

Following clinical practice, the data analysis was performed based on clinical expert ratings and was thus investigator-dependent. Therefore, this kind of clinical data analysis is prone to rater bias. For that reason, all clinical raters were blinded with respect to the identity of the EEGs of interest. For this reason, we can reject the suggestion that a rating bias might have increased the prevalence of pathological EEG findings in our BPD group.

Further, all EEGs deemed to be pathological were ultimately assessed by a team of three experts, including one board certified consultant neurologist (SB) and another very experienced board certified consultant neurologist and epileptologist from the local university epilepsy center (DMA). Therefore, we think that the quality of the clinical rating of the EEGs corresponds to the highest clinical standards. However, since the first line trained rater (MF) is not an experienced epileptologist, we cannot rule out the fact that false negative EEG ratings might have lowered the prevalence of pathological EEG findings. In further studies, an automatic and quantitative means of detecting IRDAs and IRTAs should be performed to verify our results.

The sample size could have been larger than the 96 patients we decided to include in this project. However, since visual EEG analysis in general and the algorithm of analysis we chose in this study in particular is extremely time consuming, for practical reasons we were not able to create a larger sample. Also, the size of our sample compares well to publications in the literature, as can be deduced from Table 4.

The way we defined our sample does have an impact on the generalizability of our findings. In order to avoid an overestimation of the rate of EEG pathologies, we excluded patients with any kind of neurological comorbidity as well as those with a history of birth complications, febrile seizures, a history of meningitis, or encephalitis, and a family history of epilepsy. All of these factors could have contributed to pathological EEG findings and were therefore excluded. We were not able to exclude a medication effect in the overall sample but, as mentioned above, the rate of EEG pathologies in the unmedicated BPD patients was the same as that in the mixed group. Since in the general medical settings quite a few BPD patients do fulfill at least one of the exclusion criteria mentioned above, we cannot generalize our findings to this clinical sample.

Also, in order to simulate the classical clinical diagnostic setting, we assessed only routine EEG studies of 20 min length. Repeated and prospective EEG studies, including sleep and sleep deprivation recordings, would most likely have produced higher prevalence rates of pathological findings, possibly including classical epileptiform activity. Therefore, our detection rate of EEG abnormalities has to be regarded as a minimum detection rate and it is very likely that the prevalence of pathological EEG findings will be even higher if such exclusion criteria are not applied or repeated and more elaborate assessment methods are applied.

Despite these limitations, we here present the largest study to date into possible EEG pathologies in BPD patients and produce evidence that a considerable subgroup of 14.6% of BPD patients do display EEG pathologies in terms of IRDAs or IRTAs. Thus, a question arises as to what this might mean from a pathophysiological point of view and with respect to treatment.

### Clinical relevance of IRDAs/IRTAs in BPD

**Figure 2:**
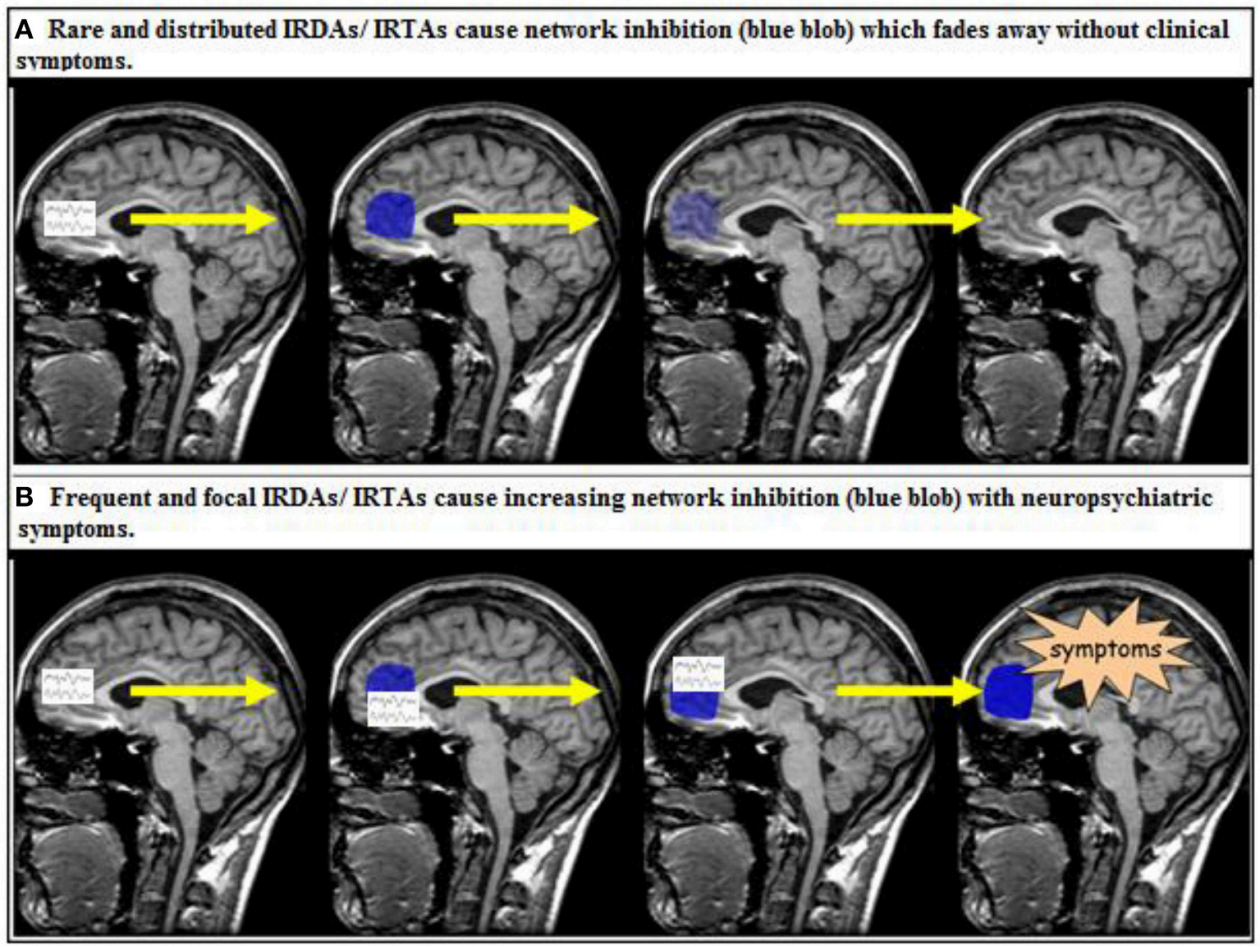
**The local area network inhibition (LANI) model as a potential paraepileptic pathomechanism (Tebartz van Elst et al., 2011; Tebartz van Elst and Perlov, 2013)**. IRDA, intermittent rhythmic delta activity; IRTA, intermittent rhythmic theta activity.

The clinical relevance of intermittent rhythmic delta or theta EEG activity is poorly understood. As discussed in the introduction, both IRDAs and IRTAs are clearly regarded as pathological EEG patterns (Brigo, 2011). However, it is unclear precisely what their presence means in terms of disturbed neuronal information processing. Clearly, none of our patients do suffer from epilepsy. The LANI hypothesis outlined in the introduction is a pathophysiological model that can explain why patients exhibiting IRDAs or IRTAs may develop clinical neuropsychiatric symptoms at one time (i.e., when IRDAs are focal and frequent enough to stimulate above threshold reactive neuronal network inhibition) but not at another time (i.e., when IRDAs are distributed or rare so that reactive neuromodulation remains subthreshold and can fade away before another IRDA event occurs) (Tebartz van Elst et al., 2011). If true, the IRDA/IRTA model would be a model of a paraepileptic pathomechanism, i.e., a pathophysiology in which non-ictal, but paroxysmal EEG activity results in neuropsychiatric symptoms via reactive network modulation (Figure 2). It is important to stress that this assumed mechanism cannot be regarded as epilepsy (Tebartz van Elst et al., 2011). Another well-established example of such a paraepileptic pathomechanism is that of Todd's paresis (Fisher and Schachter, 2000), where epileptic seizure activity also induces neuromodulatory processes that result in long-term functional alterations of the neurophysiology and affected neuronal networks (Fisher and Schachter, 2000; Schulze-Bonhage and Tebartz van Elst, 2010; Tebartz van Elst et al., 2011). However, in Todd's paresis the diagnosis is necessarily linked to classical seizures whereas the LANI hypothesis is based on non-ictal paroxysmal activity acting as the trigger mechanism, which means that a diagnosis of epilepsy cannot be made (Tebartz van Elst et al., 2011).

The idea that non-ictal epileptiform activity in the brain causes secondary modulation of the neurophysiology of neuronal networks, which in turn causes psychiatric symptoms, is not new to neuropsychiatry (Stevens, 1988, 1995, 1999; Bruton et al., 1994; Heimer, 2000, 2003; Tatlidil, 2000; Läppchen et al., 2008, 2011). Such models are difficult to prove and the present study was a first step in that direction as it validated the prevalence of the EEG phenomenon of IRDAs or IRTAs in a fairly typical sample of BPD patients in the tertiary referral setting of a specialized center offering specific therapy.

At the end of the day, the question of the precise pathophysiology and meaning of IRDAs and IRTAs is a rather academic matter and it will take a while until we are able to clarify it. However, from a clinical point of view, another more important therapeutic question arises, i.e., whether or not the observation of such EEG pathologies in BPD patients might have therapeutic implications. We recently described a patient with IRTAs and severe dissociative and auto-aggressive symptoms who responded very well to therapy with valproate as well as topiramate later in the course (Tebartz van Elst et al., 2011). Based on this observation, one might speculate that the presence of IRDAs or IRTAs in BPD patients might be a biological predictor of a positive response to anticonvulsive treatment options. To our knowledge, there are no studies published in the literature focusing on this question. However, the answer to this question might be very important for the therapy of a subgroup of BPD patients. Clearly, further research is needed to resolve this important clinical issue.

## Summary

To our knowledge, this uncontrolled study is the largest survey to date on the prevalence of EEG pathology in a carefully selected, retrospectively defined sample of BPD patients where all possible risk factors for pathological EEG findings led to exclusion. The EEG readings followed high standards and were conducted by blinded highly qualified raters. We found a prevalence of IRDAs or IRTAs in 14.6% of BPD patients. Further research is needed to clarify whether or not such pathological EEG findings might predict the response to anticonvulsive pharmacotherapy in these patients.

## Author contributions

All authors listed, have made substantial, direct and intellectual contribution to the work, and approved it for publication.

### Conflict of interest statement

The authors declare that the research was conducted in the absence of any commercial or financial relationships that could be construed as a potential conflict of interest.The reviewer, XL, and handling Editor declared their shared affiliation, and the handling Editor states that the process nevertheless met the standards of a fair and objective review.
